# The Legal Status of Crown and Bridge Work

**Published:** 1888-02

**Authors:** Solomon J. Gordon, John K. Beech

**Affiliations:** 833 Broadway, New York City; 9 Law Chambers, New Haven, Conn.


### The Legal Status of Crown and Bridge Work.

In order that the readers of the REGISTER may understand the present legal conditions of the patents pertaining to Crown and Bridge Work, the following communication is given. This is clear and understandable, and will show at a glance what may and what may not be done, as affairs now stand.

*A. L. Northrop, D.D.S.*

DEAR SIR:--In answer to your request on behalf of first District Dental Society of New York, asking for our opinion as to the legal position of the dental profession, with regard to the crown and bridge patents of the "International Tooth Crown Company," in view of the recent decision of Judges Wallace and Shipman, in the Richmond and Gaylord suits, and advice as to relief from further claims made under the Low Bridge patent, we have to say.

These suits involve the validity of the two patents to Cassius M. Richmond, Nos. 277,941 and 277,943, for "Tooth Crowns, etc.," the patent to Alvin S. Richmond, No. 277,933, for "bridge," all dated May 22d, 1883, and the patent of James E. Low, for "method of supporting artificial teeth by bands cemented to permanent teeth," No. 238,940, dated March 15th, 1881.

The first two patents cover what is known as the "Richmond" and the "Sheffield" tooth crown in all its varieties. They were held invalid, and therefore you are at liberty to make such tooth crowns without being in any way liable to the International Tooth Crown Company.

The complainants have appealed this case to the U. S. Supreme Court, but we do not advise you that any different decision will probably result. The practical result is that the tooth crown is free.

The patent for the Richmond bridge was also held invalid, but the Low patent was declared to be good. This Low patent covers a bridge attached to continuous bands cemented to adjoining permanent teeth, "whereby said artificial teeth are supported by said permanent teeth without dependence on the gum beneath."

The Richmond patent is, as you will remember, for a bridge supported by caps, and the Court held that it was not invention for Richmond to support a bridge on caps, but it was invention for Low to support a bridge on bands, taking all the surrounding circumstances into consideration, and that as a cap was nothing but a band with a roof on it, the Richmond bridge infringed the Low patent.

The practical effect of this decision, if the complainant chooses to follow it up diligently, and unless some new evidence is found, will be to shut the profession out from inserting permanent bridges supported at one or more points by cemented caps or bands without dependence on the gum.

As the matter now stands, any dentist inserting a Richmond bridge (according to the decision), infringes the Low patent; and an injunction would doubtless now be granted by any Federal Judge on application, on the strength of that adjudication alone.

An appeal can be taken by the defendants to the Supreme Court, a year or so hence, after an accounting by them, and determining the amount of profits or damages the complainant is entitled to recover.

The way of relief is for all the dentists of the United States, who supported artificial teeth on a band or bar, surrounding and extending between permanent teeth prior to September, 1878, to send to us at No. 833 Broadway, New York City, or to No. 9 Law Chambers, New Haven, Connecticut, a truthful description of what he did, and for whom, and where and when.

If such proofs can be made strong and clear enough to satisfy the Court that what Low described was well known, and had been long practiced by dentists in the United States before Low claims to have done it, the present case might be opened for re-hearing on the newly discovered evidence—or the Courts might refuse to grant injunctions, upon the ground that the present decision would have been the other way if this evidence had been before it—at any rate, the question of the validity of the Low patent would be re-tried, if its owner ever had the temerity to sue a dentist whose mouth had not been closed by a license, in which he covenanted never to deny its validity.

Whether, in a suit against such a licensee, the Court would enjoin upon the *covenants*, under a patent declared void, either before or after the taking of the license, we can not say.

Your obedient servants,

SOLOMON J. GORDON,
833 Broadway, New York City.
JOHN K. BEECH,
9 Law Chambers, New Haven, Conn.

